# Effect of equiaxial cyclic strain on cardiomyogenic induction in mesenchymal stem cells

**DOI:** 10.1007/s40204-018-0102-5

**Published:** 2018-10-26

**Authors:** Nasim Rezaee, Mohammad Tafazzoli-Shadpour, Nooshin Haghighipour

**Affiliations:** 1 Faculty of Biomedical Engineering, Amirkabir University of Technology, 424 Hafez Ave, Tehran, Iran; 2 Cardiovascular Engineering Lab, Faculty of Biomedical Engineering, Amirkabir University of Technology, 424 Hafez Ave, Tehran, Iran; 3 National Cell Bank of Iran, Pasteur Institute of Iran, 69 Pasteur Ave, P.O. Box: 1316943551, Tehran, Iran

**Keywords:** Mesenchymal stem cells, Equiaxial strain, Cardiomyogenic differentiation, Cytoskeleton, Gene expression

## Abstract

Differentiation of stem cells and functionality of target cells are regulated by microenvironmental stimuli to which the cells are exposed. Chemical agents such as growth factors and physical parameters including mechanical loadings are among major stimuli. In this study, equiaxial cyclic strain with two amplitudes was applied on rat adipose-derived mesenchymal stem cells (rAMSCs) with or without 5-azacytidine. The mRNA expression of cardiac-related genes was investigated through RT-PCR (polymerase chain reaction) method. Moreover, morphological features and the actin structure of the cells were studied. Results were indications of significant increase in mRNA expression among four target genes, which marked the increase in two principal cardiac markers of GATA4 and α-cardiac actin, and lesser increase in two other genes (NKX2-5, βMHC) in all experimental groups treated chemically and/or mechanically. Such effect was maximal when both treatments were applied describing the synergistic effect of combined stimuli. All treatments caused significant increase in cell area and cell shape index. The well spreading of cells was accompanied by enhanced actin structure, especially among samples subjected to mechanical stimulus. Both effects were among required features for functional muscle cells such as cardiac cells. It was concluded that the cyclic equiaxial strain enhanced cardiomyogenic induction among rat adipose-derived mesenchymal stem cells and such effect was strengthened when it was accompanied by application of chemical factor. Results can be considered among strategies for cardiomyogenic differentiation and can be employed in cardiac tissue engineering for production of functional cardiomyocytes to repair of damaged myocardium.

## Introduction

Full disability of tissue or its loss is among destructive medical conditions which require expensive and time-consuming treatment strategies. Myocardial infarction is a permanent and mainly irreversible cell death in part of the cardiac tissue. Past treatment strategies such as use of organ transplants, mechanical devices and implants have faced limitations (Haraguchi et al. 2012). For example, therapies such as angioplasty are partly capable of temporary termination of heart attacks, but are not able to reconstruct the damaged tissue (Zhou et al. 2006). Emerging approaches such as tissue engineering and cell therapy have shown new possibilities for the treatment of damaged tissues through natural re-growth or repair.

Mesenchymal stem cells (MSCs) are capable of self-renewal and differentiating into other cell types (Pountos et al. 2007). For cardiac treatment, MSCs have been used for in vitro differentiation into beating cardiomyocytes (Haraguchi et al. 2012), in vivo repair of myocardium (Vidarsson et al. 2010), and myocardial infarction therapy (Fisher et al. 2015).

Cells of human body are subjected to a dynamic environment by which their function within the state of homeostasis is defined. Exploring the microenvironment of MSCs provides a better understanding of differentiation towards functional cells and consequently reconstruction of damaged tissues. This has led scholar research to provide similar environmental conditions and stimuli during in vitro differentiation of stem cells to obtain well-functioning target cells. Current methods that are employed for the differentiation of stem cells involve the use of biochemical agents, physical factors such as mechanical, electrical, thermal and electromagnetic stimuli, and co-culture with other cells (Maul et al. 2011; Kavand et al. 2016; Pires et al. 2015).

Besides biochemical factors, mechanical stimuli such as shear (Gholami et al. 2016) and tensile (Park et al. 2004) stresses have been shown to influence stem cell behavior. They strongly affect stem cell structure, gene expression and protein synthesis, and consequently stem cell function and fate (Keung et al. 2009). Due to the involvement of various biological and physical parameters, in vivo studies for assessment of the effect of mechanical stimuli on cells are rather impractical. Hence, in vitro studies have been vastly utilized to investigate the effects of mechanical loadings on different cell types including MSCs (Wang and Chen 2013). Various types of loading have been shown to influence the biological behaviors of MSCs under certain conditions, depending on the in vivo mechanical microenvironment that the targets tissues experience (Ghazanfari et al. 2009). For instance, since pulsatile blood pressure exerts tensile strain on the vessel wall in circumferential direction, cyclic uniaxial strain has been shown to be more effective in differentiation of MSCs into smooth muscle cells compared to cyclic equiaxial strain (Park et al. 2004). Further experiments have shown that hydrostatic pressure and hydrodynamic shear stress contribute to the differentiation of stem cells into chondrocytes (Wagner et al. 2008)and osteocytes (Knippenberg et al. 2005), respectively. Since myocardial cells are exposed to the longitudinal and radial strains, we hypothesized that equiaxial strain may contribute to cardiomyogenic induction in MSCs through elevation in expression of cardiac-related genes.

Different strategies for differentiation of stem cells into cardiomyogenic lineage have been proposed (Heng et al. 2004), mostly based on the biological conditions that myocardium experiences in vivo, such as using various growth factors (Sachinidis et al. 2003), synthetic chemicals (Fukada 2001), free radicals and reactive oxygen species (Sorescu and Griendling 2002), and co-culturing stem cells with other cells (Mummery et al. 2003). It was further shown that cyclic uniaxial stretch upregulated protein synthesis in adult cardiomyocytes (Wada et al. 1996), and shear stress triggered cardiomayogenic differentiation that corresponds to expression of specific protein markers (Huang et al. 2010). Furthermore, it was shown that cyclic strain can differentiate rat bone marrow mesenchymal (rBMSCs) cells into cardiomyocytes (Huang et al. 2012), and that application of cyclic strain with 5-azacytidine can result in enhanced differentiation compared to the application of shear stress.

The conventional treatments in the field of cardiovascular diseases are expensive and lag reconstruction of damaged tissue. Such shortcomings are crucial in myocardial infarction, which is the main cause of world mortality. Hence, cell therapy approaches and regenerative medicine can be considered as alternative methods by which the original tissue is repaired. Such methods require functional cells, not only to be able to survive in target tissue, but also to stimulate tissue reconstruction. Since myocardium is subjected to equiaxial stretch caused by ventricular pressure, it is a proper strategy to engineer mesenchymal stem cells through exposure to such tension during cardiomyogenic induction. Hence to further evaluate the effectiveness of new methods to induce cardiomyogenic differentiation among MSCs, and to obtain a better insight of the role of mechanical stimuli in cardiovascular development and remodeling, here we investigated the effects of equiaxial cyclic strain on cardiomyogenic fate of rat adipose-derived mesenchymal stem cells (rAMSC). Despite other types of mechanical stimulations, the effects of equiaxial tension on differentiation of AMSC have not been yet studied. MSCs were isolated from rat fat tissues and equiaxial cyclic strain was exerted on cells in the presence or absence of chemical stimulus (5azacytidine chemical compound). We specifically addressed whether equiaxial cyclic stretch with different amplitudes contributes to the expression of cardiomyogenic markers and investigated the possible synergy between mechanical and chemical stimuli in this matter.

## Materials and methods

To study the effects of cyclic equiaxial strain on cardiomyogenic differentiation of rAMSCs, the expression of cardiomyogenic markers and morphological features of cells among test and control groups were examined. Cells with no chemical or mechanical treatments were cultured as the control group with the same culture duration of test samples. Test groups included two groups of cells subjected to cyclic equiaxial strain with two amplitudes of 5% or 10%, cells treated by chemical factor (5azacytidine), and finally samples subjected to 10% cyclic equiaxial stretch accompanied by chemical factor (5azacytidine). Regardless of the type of stimulation, all test samples were treated for 24 h as previously reported for continuous mechanical treatment of cells (Huang et al. 2012). It has been suggested that that longer durations might damage cells, while shorter times might not induce sufficient target differentiation. Samples of test groups were treated for 24 h and expression of cardiac-related markers was studied after 1 week of further culture of treated cells (Huang et al. 2012). The 1 week interval was to evaluate long-term upregulation of genes, since in some occasions, after initial upregulation, gene silence occurs a few days after cell conditioning (Huang et al. 2010). Additionally, cell images were captured before and after treatments and morphological analysis was performed through image processing.

### Cell preparation and culture

The abdominal fat tissue of 5-week male rat (Pasteur Institute of Iran) was isolated in a surgical procedure and stored in sterile DMEM F12 (Sigma, USA) under sterile circumstances and approval of Ethical Committee of National Cell Bank of Iran (NCBI). The fat tissue was washed with phosphate-buffered saline (PBS) (Sigma, USA) three times and cut into small pieces. To digest enzymatically, the samples were incubated in 0.1% collagenase type I (Gibco, USA) at 37 °C and 5% CO_2_ with intense shaking every 10 min to disperse the adipose tissue effectively. After 30 min of incubation, DMEM F12 with 20% fetal bovine serum (FBS) (Gibco, USA) was added to the digested tissue to stop collagenase activity. The undigested tissue was removed and samples were centrifuged at 2000 rpm for 5 min. After further shaking, rAMSCs were separated from adipocytes by further centrifuging at 2000 rpm for 5 min. The upper medium was removed and the pellet was suspended in DMEM F12 with 10% FBS, 1% penicillin/streptomycin and 1% L glutamine in a conventional flask before incubation. After 24 h, the medium was replaced and the medium was further replaced every 3 days. When cells were 90% confluent, they were detached using trypsin (Sigma, USA) containing 0.1% EDTA (Sigma, USA) and cultured in DMEM F12 with 10% FBS. The rAMSCs in passage 3 were used for subsequent experiments.

### Experimental set up and test conditions

Medical grade poly(dimethyl siloxane) or PDMS (Sylgard^®^184, Dow Corning, USA) membranes were fabricated according to manufacturer’s suggested protocols. The two components of the pre-polymer and cross-linking agent (ratio of 10:1) were mixed and the vacuum-conditioned samples were degassed. Samples were treated at 100 °C for 1 h and membranes of 0.5 mm thick were produced and used in sterile conditions to serve as the cell substrate (Khani et al. 2015) Before cell seeding, the membranes were coated by 5 µg/cm^2^ collagen type I (Sigma-Aldrich, USA) for proper attachment. The collagen concentration was kept minimal to ensure cell adherence without surface chemical manipulation (Goli-Malekabadi et al. 2011). Cells of all control and test groups were cultured on similar membranes with the density of 4000 cells/cm^2^. Control samples were incubated in 5% CO_2_ and 37 °C without exposure to external stimuli.

**Fig. 1:**
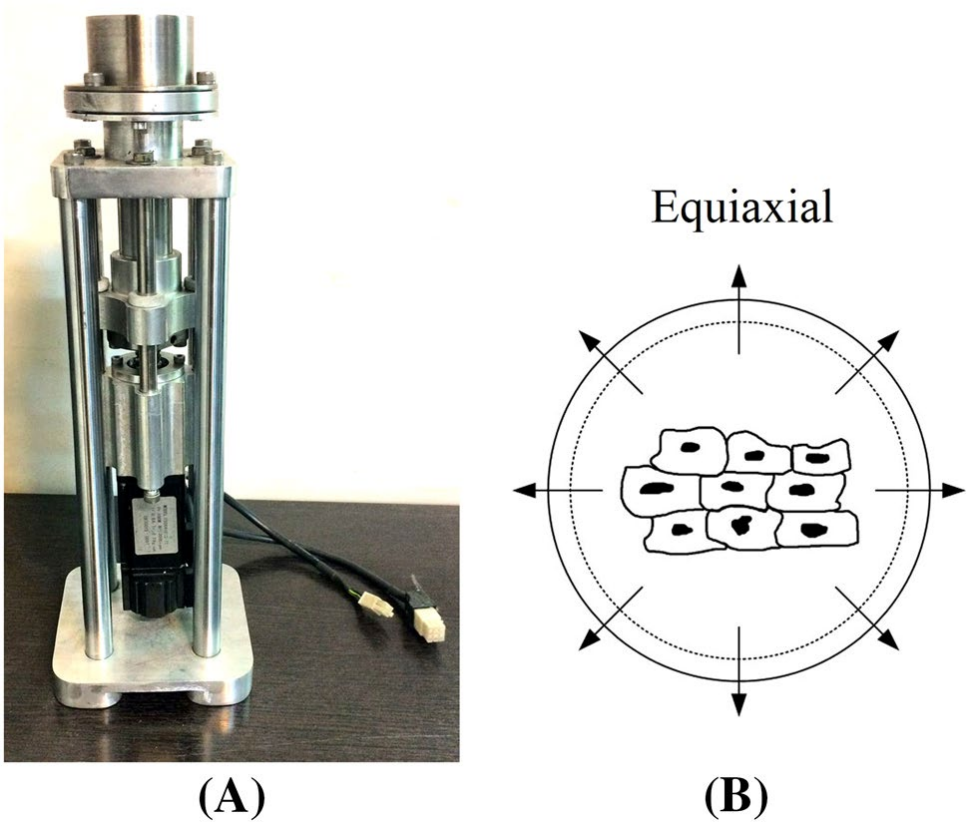
Mechanical stimulation device: **a** equiaxial strain is exerted on the cells through the vertical post located beneath the medium container with the cells cultured on the membrane, **b** equiaxial strain schematic

To apply cyclic equiaxial stretch on rAMSCs, a custom-made apparatus was designed and manufactured capable of working inside an incubator with the strain and frequency range of 0–25% and 1–3 Hz, respectively. The device consisted of electrical and mechanical units. The electrical unit included a servo motor, power supply and a custom-made human–machine interface for adjustment of the experimental setup. The mechanical unit included couplings, ball-screw supports, guides, gripper and the medium container (Fig. 1). A post beneath the surface of the flexible membrane was used to create planar strain in the membrane during the downward movement of the medium container. Cells subjected to mechanical loading were subjected to either 10% or 5% cyclic equiaxial tensile strain for 24 h with the frequency of 1 Hz, comparable to frequency of the beating human myocardium.

To study the behavior of rAMSCs treated by biochemical stimulus and compare the results to those of samples subjected to mechanical loading, the cells were treated by medium containing 5-azacytidine (10 µmol/L) (Sigma-Aldrich, USA) for 24 h. Finally, the samples were exposed to both chemical factor and mechanical loading (10% amplitude and 1 Hz frequency) for 24 h to examine the possible synergistic effects of both stimuli. Despite using strain values of 5% and 10% for mechanically treated groups, we only used the 10% amplitude for samples treated by both stimuli. This value was selected after finding an optimized value by which the effects of mechanical stimulation were maximized. Hence to examine the synergistic effect of mechanical and chemical stimuli, we utilized the 10% strain amplitude.

### Analysis of cardiomyogenic induction

The expression of cardiac genes was investigated to analyze cardiomyogenic induction among test groups using RT- PCR method. After treatment of samples, they were further cultured for 7 days (Huang et al. 2012). Then, the total RNA of the five experimental groups was extracted using RNeasy Plus mini kit (Qiagen, USA). A QuantiTect Reverse Transcription kit (Qiagen, USA) was used for cDNA synthesis from 1 g of total RNA of each sample. The expressions of four target genes, GATA4, α-CA, NKX2-5, βMHC, and β-actin as the reference gene, were quantified in triplicate using SYBR Green. Master Mix and ABI StepOne RT-PCR (both from Applied Biosystems, USA) were utilized and the primers were designed by Primer Express software (version 3). The primer sequences used in RT-PCR analysis are shown in Table 1. The gene expression was quantified using comparative threshold (Ct) method (2^−∆∆ct^). The Ct values of target genes were normalized to those of β-actin and the resultant values were then normalized to the values of the control group.

**Table 1:** Primer sequences of specific and non-specific cardiac markers used in RT-PCR analysis

Names	Sequences	PCR product (bp)
Alpha-cardiac actin (α-CA)	5ʹ-ACTCCTATGTAGGTGACGAGGC-3ʹ	337
Gata4	5ʹ-GACGTTATGAGTCACACCGTCG-3ʹ	208
NKX-2.5	5ʹ-AGAAGGCAGAGAGTGTGTCA-3ʹ	178
βMHC	5ʹ-CAGTGTGGTGGTGGTAGTCT-3ʹ	196
βACTIN	5ʹ-ACCCTCGGGCGGATAAGAA-3ʹ	226

Among cardiac markers, α-CA is the major protein of the thin filaments in cardiac sarcomeres, which is responsible for muscle contraction and generation of force to support pumping function of the heart (Orban et al. 2008). GATA4 is a vital cardiac marker which is shown to regulate genes involved in embryogenesis and myocardial differentiation and function (Orban et al. 2008; Latinkic et al. 2002). This marker is a transcriptional mediator which responds to mechanical force, such that the direct stretching of the ventricles activates GATA4 (Hautala et al. 2002). An elevation of GATA4 activity is thought to induce angiogenesis in infarcted heart tissues (Heineke et al. 2007). This marker functions in combination with other essential cardiac transcription factors such as Nkx2-5 which is crucial in cardiac differentiation and normal growth of the embryonic myocardium. Finally βMHC is a gene encoding a myosin heavy chain beta (MHC-β) isoform that is primarily expressed in the heart (Uhlen et al. 2010, 2015).

### Actin staining

Actin staining was performed on the cytoskeletal actin structure of rAMSCs of the test and control groups. First, the medium was removed and cells were washed three times with PBS before fixation with 3.7% paraformaldehyde for 20 min. The cells were then permeablized using 0.1% Triton X-100 for 15 min. After washing cells with PBS, they were incubated with 4 μg/mL Phalloidin solution in PBS for 45 min in dark environment. The cells were then rinsed with PBS and the actin fibers were detected by fluorescence microscope (Zeiss, Germany).

### Image processing and topological analysis

Before and after treatments, cell images from control and test groups were captured by an inverted microscope (Zeiss, Germany) and digital camera (Sony, Japan). Images were transferred to ImageJ2 image processing software (USA) and then morphological parameters of cell area and cell shape index (SI) were obtained. The latter was calculated as:

1 $${\text{SI}} = \frac{1}{N}\sum\limits_{i = 0}^{N} {\frac{{4\pi S_{i} }}{{P_{i} }}}$$

in which *N* indicates cell number, *P* is the cell perimeter, and *S* is the cell area. The shape index is inversely related to cell elongation (Owatverot et al. 2005).

### Statistical analysis

All tests were repeated at least three times for statistical verification. For morphological analysis, for each test at least 5 images were taken and in each image, morphological parameters of at least 20 cells were calculated. Data were presented as Mean ± SD. To statistically compare results of test groups, multi-factorial one-way ANOVA followed by post hoc Tukey’s honest significant difference (HSD) analysis was performed assuming significance set at *P* < 0.05. To further compare results of each test group to those of control group, *t* test analysis was carried out.

## Results

### Morphological analysis

**Fig. 2:**
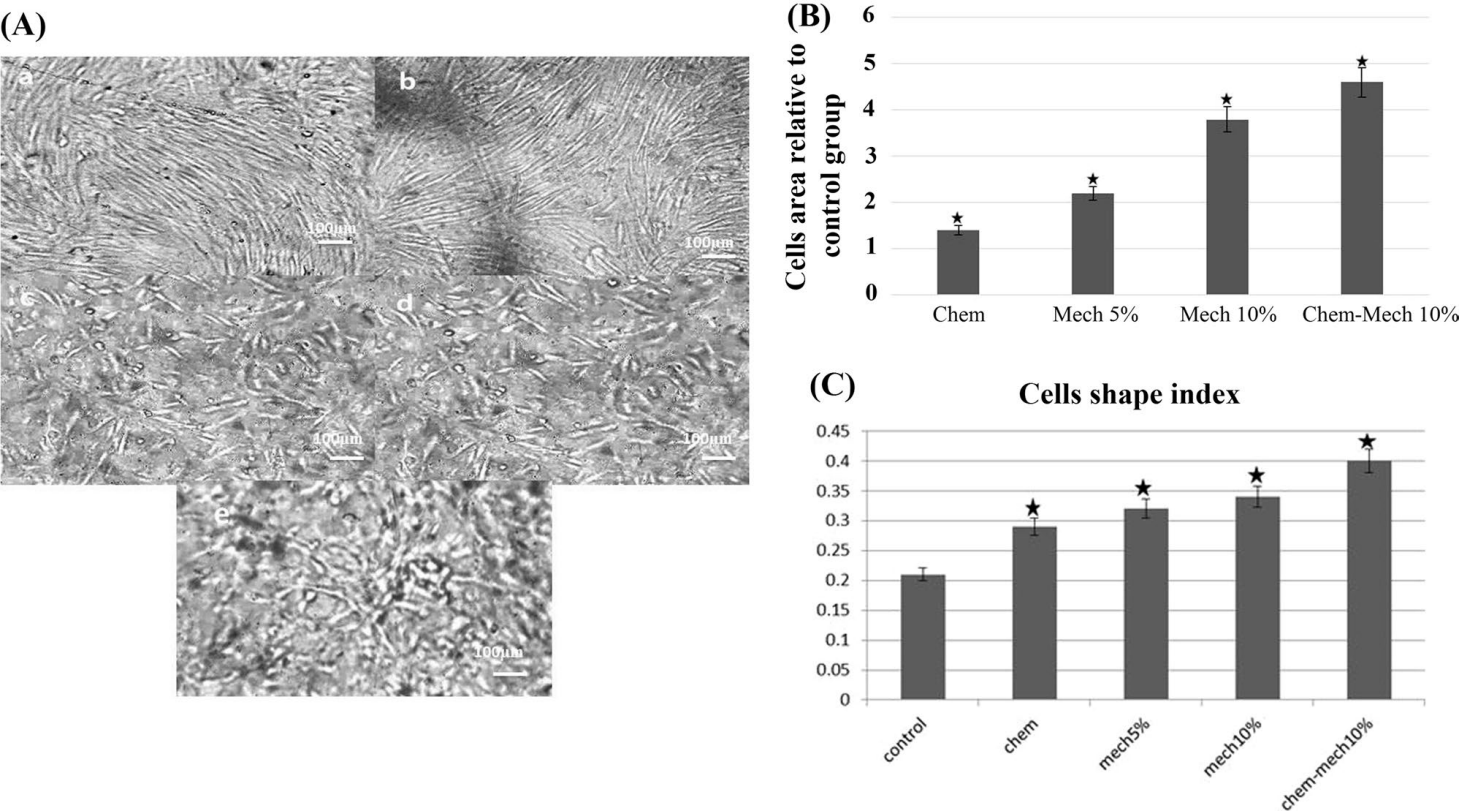
Cell images in experimental groups (**a**) control, (**b**) treated by chemical factor, (**c**) treated by 5% stretch, (**d**) treated by 10% stretch, (**e**) treated by mechanical and chemical stimuli (100×), **b** cell area. The average values of cell area among test groups were calculated and presented as relative to that of control group, **c** comparison of cells shape index in experimental groups [*shows significant difference compared to the control group (*P* < 0:05)]

Figure 2a shows cell images of control and test groups in which morphological alterations in area, perimeter and elongation are observed. Figure 2b, c indicates quantified morphological features of cell area and shape index for all control and test groups. Cell area increased for all test samples after chemical, mechanical and concurrent treatments. Compared to control group, such increase was significant among mechanically treated samples (5%, 10%) and samples exposed to mechano-chemical treatment (*P* < 0.05). When cell area was compared among all test groups, a statistical difference was indicated by one-way ANOVA (*P* < 0.05). Furthermore, cell shape index increased in all experimental groups relative to control group. In other words, all cells were less elongated after treatment and this was statistically significant for all treated samples (*P* < 0.05). Followed by ANOVA, further post hoc analysis revealed significant differences between morphological features of the group treated chemically with the group treated by both stimuli; however, such difference was not observed between mechanical and mechanical-chemical groups. This describes the defined effect of mechanical stimulation on morphological features of cells.

### Gene expression

**Fig. 3:**
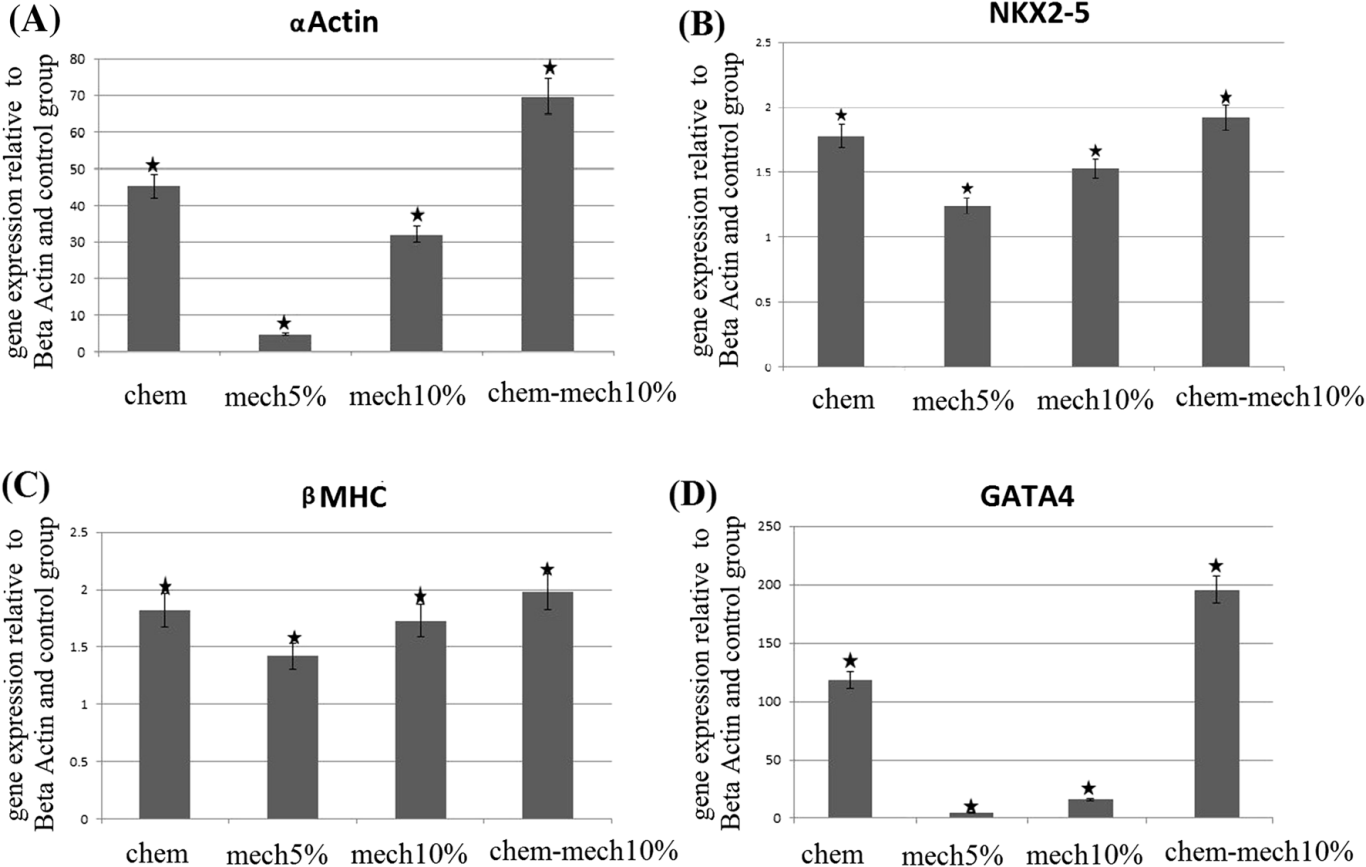
Effects of four types of 24 h treatments on the cardiac-related genes expressions: mRNA expression levels of α-CA, NKx2.5, βMHC and GATA4 were assessed on the 7th day by quantitative RT-PCR analysis. The expression of each gene was relative to the expression of β-Actin and then normalized to that of control group. **a** α-CA **b** NKx2.5 **c** βMHC **d** GATA4. Results are shown as mean ± SD (*describes significant difference with the control group)

The expression levels of the major cardiac markers were determined on the 7th day post treatment. Figure 3 describes results of the quantified expression levels of 4 cardiac specific genes of α-CA, GATA4, NKX-2.5, βMHC relative to β-Actin (house-keeping gene) normalized to that of the control group.

The expression of cardiac-related genes increased among all test groups, although such increase was markedly higher for GATA4 and α-CA compared to NKX-2.5 and βMHC (Fig. 3). Results revealed that mechanical strain is a potent enhancer of cardiomyogenic induction even without treatment by chemical factor, although a marked synergistic effect was observed when both mechanical and chemical stimuli were applied (Fig. 3). Among samples treated solely by mechanical loading, stronger expression of all markers was observed for samples subjected to 10% strain compared to those treated by 5% strain. In general, treatment by the chemical factor showed higher effect on cardiomyogenic induction in rMSCs than mechanical treatment alone for all target genes (*P* < 0.05 for GATA4 and α-CA). However, a combination of cyclic strain and 5-aza had strongest effect on mRNA expression of all target genes than either treatment alone by the chemical factor or mechanical strain (*P* < 0.05 for GATA4 and α-CA) (Fig. 3).

GATA4 showed the highest expression level among target genes for all treated samples. Quantification of genes expression in comparison to control group showed GATA4 mRNA levels increased by 4.7, 16, 118.2 and 195.5 folds in average of 3 tests among samples treated by 5%, 10% strain, chemical and mechano-chemical stimuli, respectively (*P* ≪ 0.05). A similar trend with distinct increase in expression was observed for α-CA marker with corresponding 4.8, 32.1, 45.2 and 69.7 folds increase (*P* ≪ 0.05). The elevation of gene expression for the other two markers was noticeably lower for all treatments although the trend of increase in gene expression was similar to the first two markers as 1.24, 1.53, 1.78 and 1.92 folds increase for NKX-2.5 and 1.42, 1.73, 1.82 and 1.98 folds increase for βMHC accordingly (*P* < 0.05).

Results of post hoc analysis for the expression of two markers, GATA4 and α-actin revealed strong difference between expressions of those markers among all test groups (*P* < 0.05). For the other two markers, results did not show significant differences between the test groups of chemical treatment with those of both mechanical treatments. However, significant differences were found between mechanical groups with mechanical–chemical groups.

### Actin structure

**Fig. 4:**
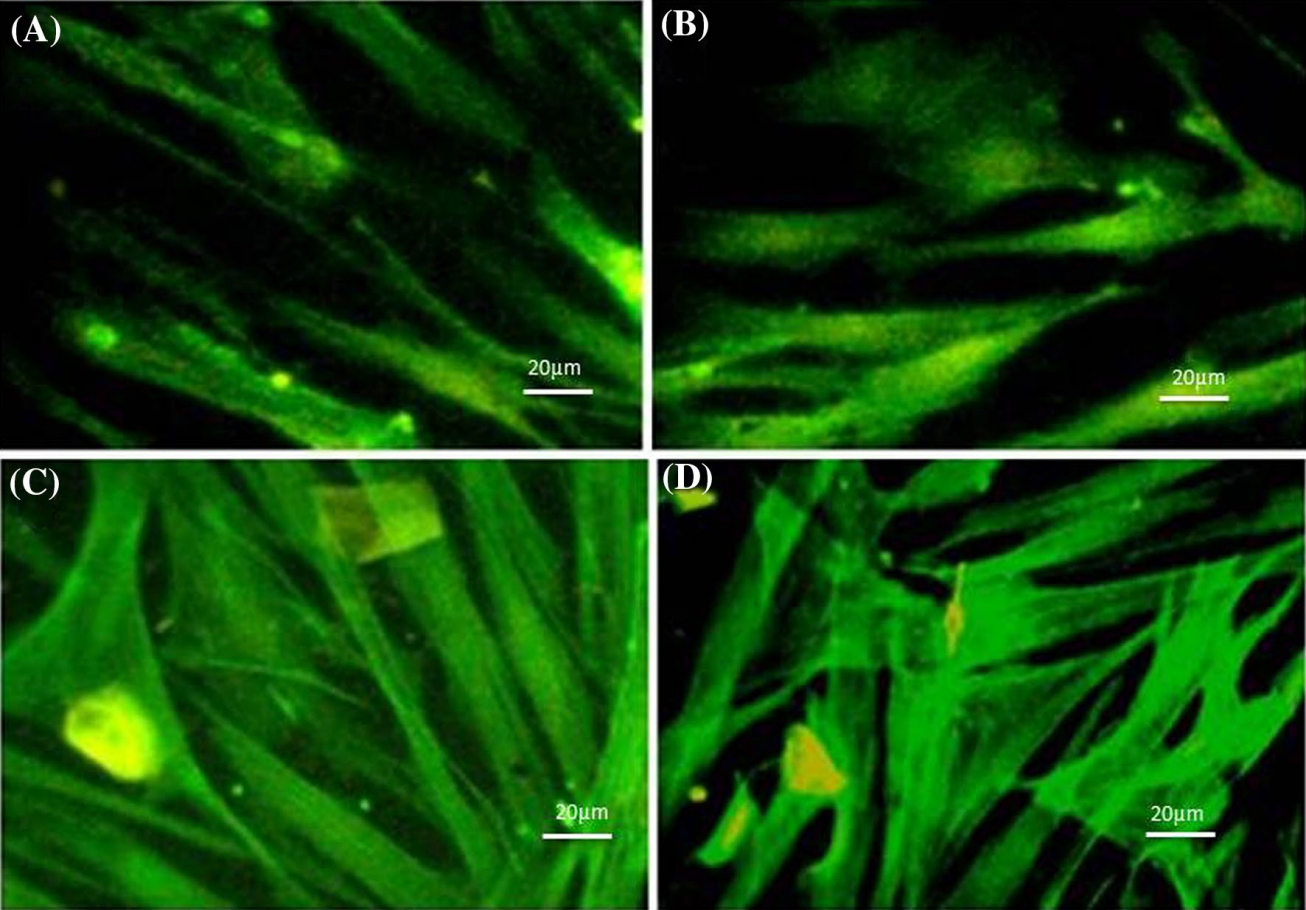
Actin structure of rAMSCs through phalloidin staining of experimental groups (200×): **a** control samples, **b** samples treated by chemical agent, **c** samples treated by 10% strain and **d** samples treated by both stimuli

Following actin staining, the resultant images indicated influence of mechanical loading on actin arrangement and alignment of rAMSCs (Fig. 4). The cells treated by both chemical and mechanical stimuli responded with altered cytoskeleton while expressing cardiac-specific genes. In general, mechanical stimulation resulted in a strong arrangement of actin fibers, while such effect was less visible among samples treated by chemical factor. Inclusion of mechanical loading during treatment of the rAMSCs with chemical factor resulted in alignment of cytoskeletal actin and generation of stress fibers which are essential features in the behavior of muscle cells such as cardiomyocytes.

## Discussion

Stem cell differentiation is influenced by the environmental conditions in which target cells experience in vivo (Keung et al. 2009). Depending on the microenvironmental stimuli in which stem cells are exposed to, the function of the target cells is regulated. Such phenomenon is particularly fundamental when muscle cells such as cardiomyocytes are required with optimized contractility and force generation for the repair of damaged myocardium. Considering biological tissues exposed to mechanical loading within the human body, many studies have explored the positive effect of different loading types on the differentiation of stem cells for cardiac tissue engineering. For instance, it was shown that cyclic compressive strain (10% or 15% at frequency of 1 Hz) enhanced chondrogenic differentiation of MSCs (Campbell et al. 2006; Martinez and Kofidis 2011), or uniaxial strain (1 Hz, 4% or 8%) elevated the mRNA level of early cardiac genes (Cx43, protein-2) in BMSCs, and finally shear stress caused cardiomyogenic induction (Huang et al. 2010). However, fewer researches have been dedicated to the effect of cyclic planar strain. The myocardial tissues are exposed to cyclic blood pressure leading to pulsatile planar stretch of the ventricular wall. Hence, the equiaxial cyclic strain is most similar loading conditions as mechanical stimulation of stem cells for cardiomyogenic induction. In current study, such loading regime significantly elevated the mRNA expression of target genes without 5-azacytidine (*P* < 0.05). Correspondingly previous results on the effects of applying 10% uniaxial cyclic strain for 24 h and culturing for 7 days on MSCs showed significant increase in expression level of GATA4, βMHC and NKX2-5 (Huang et al. 2010, 2012).

Among all treatments, the combination of equiaxial cyclic strain with 5-azacytidine caused strongest enhancement among examined cardiac genes. Such effect is in accordance with previous research that the level of GATA4, βMHC and NKX2-5 reached its highest value when subjected to 10% uniaxial strain combined with chemical factors (Guo et al. 2011). Such phenomenon confirms the synergy between both mechanical and chemical stimuli through different biological pathways. Previously we have shown synergistic effects of other types of mechanical and chemical stimuli on differentiation of mesenchymal stem cells. We have also confirmed synergistic effects of planar stretch and azacytidine on GATA4 expression of mesenchymal cells (Amin et al. 2014). Here, we further confirmed such synergy among a wide range of expression of genes and other biological parameters.

Chemical stimulation is sensed through specific biological cascades. Cells respond to the biochemical changes in extracellular matrix (ECM) through the crosstalk between integrins and cytoskeleton. Chemical signals are connected to the local adhesion sites or by regulating global cellular processes through chemical factor receptor signaling pathways (Kim et al. 2011). The pathway of mechanical stimulation is somehow different as mechanical loading is initially received by the adhesive membrane proteins and transferred to the cell body. Mechanotransductive pathways such as Wnt and TGFβ can be activated through alterations to the micro-mechanical environment (Si and Kang 2006). In addition to such pathways, mechanical incentives can be directly transferred to the cell body through the cytoskeleton with a fine mesh-like assembly of fibers. Actin structure is mostly responsible for transferring such stimulation to the nucleus and organelles (Paul et al. 2008). The remodeling in actin structure that was observed in this study is most likely related to the mechanical stimulation which in turn caused enhanced cardiac fate. Interestingly, the function of cardiac cells as muscular cells is highly dependent on the cytoskeleton. The enhanced fibrous actin network described by alignment, bundling and generation of stress fibers results in optimized muscle contractility that facilitates the function of myocardium.

Our results indicated that expressions of cardiac specific and non-specific genes were enhanced after applying equiaxial cyclic strain that is the microenvironment of cardiac cells in human body. Muscle contraction and force generation of the heart are related to activation of α-CA and are closely related to the cytoskeletal structure of cardiomyocytes (Orban et al. 2008). GATA4 is associated with the heart muscle contractility and is highly expressed in cardiomyocytes through heart development and its activation is related to healing of damaged myocardium through angiogenesis (Heineke et al. 2007). The βMHC marker is a gene encoding a myosin heavy chain beta, while the other marker, Nkx2-5 seems to be of lesser connection to cytoskeleton and physical properties of cell body, as it is more related to cardiac morphogenesis (Uhlen et al. 2010, 2015). Among the examined genes in this study, α-CA and GATA4 were shown to be markedly affected by both mechanical and chemical incentives and high variations were observed in the expression levels of these two cardiac-specific markers. This might be due to the fact that these markers are highly related to physical aspects of cell body and cytoskeleton which is sensitive to external stimuli. More specifically, equiaxial cyclic stretch, alone or in combination with chemical agent caused substantial alterations in such genes. External loading which is sensed by cells through the mechanotransductive cascades and actin structure triggers cell body remodeling and subsequently alterations in cell behavior including gene expressions.

The enhanced expression of cardiac genes results in generation of cardiac cell-like which can be utilized in cell therapy applications to reconstruct damaged myocardium. Such changes in the function of cells were further confirmed by alterations in morphology of rAMSCs which were subjected to mechanical and chemical stimuli. Previously, it was shown that when cells were subjected to uniaxial cyclic strain they were remodeled by elongation perpendicular to the loading direction most probably to minimize the strain energy and mechanical traction applied on cell bodies (Kim et al. 2011). Here, cell morphology was not affected by such phenomenon since the load was applied in all directions and did not cause cell elongation. Hence, alterations in the morphology were most probably due to change in the cell fate towards cardiac cells, especially with enhanced actin arrangement which is essential for muscular cells. While the microenvironment of the arterial wall causes circumferential tension (uniaxial loading) on vessel cells, cells of cardiac tissue within the human body are stretched planar due to ventricular pressure (equiaxial loading). Hence, such loading is a better mimic of the cardiac mechanical events. The enhanced spreading of cells caused by mechanical loading (Fig. 2) indicated a firm attachment of cells to the substrate leading to further enhancement of cytoskeletal arrangement. Interestingly, cells treated solely by chemical factor were least sensitive to cell spreading describing the marked effect of mechanical stimulation. Cyclic loading assists in cell alignment and rearrangement of cytoskeleton through bundling of actin fibers and generation of new stress fibers as described by actin staining (Fig. 4). Such effect enhances cell functionality and empowers actin structure that is specifically important for cardiomyocytes. For the muscle cells which their biological function is directly related to their cytoskeleton, the enhancement of actin structure may be of great importance when applied in vivo.

Finally, our results further confirmed the usage of adipose-derived mesenchymal stem cells (AMSCs) in tissue engineering applications. While bone marrow mesenchymal stem cells (BMSCs) have long been used in stem cell engineering, the emerging usage of AMSCs has opened new horizon in this field. This is of special interest when the abundance and easy extraction of these cells facilitate economic development of know-how in this field. It has been suggested that AMSCs may have a stronger in vitro potential in proliferation and differentiation compared to BMSCs (Martinez and Kofidis 2011), Furthermore, AMSCs might have some advantages over BMSCs in higher resistance to stressed hypoxic conditions in heart and also significant improvement in heart function and reduced infarction (Paul et al. 2013). Current studies on AMSCs also indicate increase in the mRNA level of all target genes for test groups, with stronger expression of α-CA and GATA4 for samples treated chemically–mechanically (Huang et al. 2010, 2012).

It is concluded that application of cyclic equiaxial strain with adequate characteristics enhances cardiomyogenic induction among MSCs, especially when it is accompanied by cardiac-related chemical factor. This can be considered among strategies in cardiac tissue engineering when functional cardiomyocytes are required for reconstruction of damaged myocardium.
